# Correlations between Changes in Medical Opioid Dispensing and Contributions of Fentanyl to Opioid-Related Overdose Fatalities: Exploratory Analyses from Canada

**DOI:** 10.3390/ijerph18147507

**Published:** 2021-07-14

**Authors:** Wayne Jones, Min-Hye (Angelica) Lee, Ridhwana Kaoser, Benedikt Fischer

**Affiliations:** 1 Centre for Applied Research in Mental Health and Addiction (CARMHA), Faculty of Health Sciences, Simon Fraser University, Suite 2400, 515 W. Hastings Street, Vancouver, BC V6B 5K3, Canada; wayne_jones@sfu.ca (W.J.); mhl29@sfu.ca (M.-H.L.); ridhwana_kaoser@sfu.ca (R.K.); 2 Faculty of Medical and Health Sciences, University of Auckland, 85 Park Road, Grafton, Auckland 1023, New Zealand; 3 Department of Psychiatry, University of Toronto, 250 College Street, Toronto, ON M5T 1R8, Canada; 4 Department of Psychiatry, Federal University of São Paulo (UNIFESP), R. Sena Madureira, 1500-Vila Clementino, São Paulo 04017-030, Brazil

**Keywords:** prescription opioids, mortality, synthetic opioids, nonmedical use, supply, substitution, public health, Canada

## Abstract

Canada is experiencing an epidemic of opioid-related mortality, with increasing yet heterogeneous fatality patterns from illicit/synthetic (e.g., fentanyl) opioids. The present study examined whether differential provincial reductions in medical opioid dispensing following restrictive regulations (post-2010) were associated with differential contributions of fentanyl to opioid mortality. Annual provincial opioid dispensing totals in defined daily doses/1000 population/day, and change rates in opioid dispensing for the 10 provinces for (1) 2011–2018 and (2) “peak-year” to 2018 were derived from a pan-Canadian pharmacy-based dispensing panel. Provincial contribution rates of fentanyl to opioid-related mortality (2016–2019) were averaged. Correlation values (Pearson’s R) between provincial changes in opioid dispensing and the relative fentanyl contributions to mortality were computed for the two scenarios. The correlation between province-based changes in opioid dispensing (2011–2018) and the relative contribution of fentanyl to total opioid deaths (2016–2019) was −0.70 (*t* = 2.75; df = 8; *p* = 0.03); the corresponding correlation for opioid dispensing changes (“peak-year” to 2018) was −0.59 (*t* = −2.06; df = 8; *p* = 0.07). Provincial reductions in medical opioid dispensing indicated (near-)significant correlations with fentanyl contribution rates to opioid-related death totals. Differential reductions in pharmaceutical opioid availability may have created supply voids for nonmedical use, substituted with synthetic/toxic (e.g., fentanyl) opioids and leading to accelerated opioid mortality. Implications of these possible unintended adverse consequences warrant consideration for public health policy.

## 1. Introduction

A persistent public health crisis of opioid-related overdose mortality has been unfolding across Canada since the early 2000s. Concretely, the annual total number (population rate per 100,000) of opioid-related poisoning fatalities in Canada increased from 2825 (7.8) in 2016 to 3831 (10.2) in 2019 [1,2,3]. Opioid-related fatalities occurring primarily in young- and middle-aged persons have slowed increases in population life expectancy [4]. Opioid-related mortality rates and and related dynamics in Canada have been similar to those observed in the United States [4,5].

However, there have been notable changes in the origins and pharmacological profiles of the opioids contributing to overdose mortality. While the majority of deaths in North America were related to pharmaceutical opioids a decade ago, these became gradually replaced by illicit/synthetic opioids (e.g., fentanyl/analogues, heroin) over the past decade. To illustrate: The contribution of fentanyl-type opioids to opioid-related mortality in Ontario, Canada’s most populous province, increased from 27% (2014) to 76% in 2019 [6,7]. Nationally, the proportion of non-fentanyl fatalities among total opioid-related fatalities fell from 55% in 2016 to 38% in 2019, with contributions to opioid mortality totals, however, varying substantially between provinces (ranges: 37–89% [2016]; 30–100% [2019]) [3]. This is similar to the US, where the contribution of synthetic/illicit opioids to opioid-related deaths increased from 14% in 2010 to about 65% in 2018 [1,8].

Recent studies have documented the notably amplified individual–behavioural and pharmacological overdose risk dynamics of illicit/synthetic opioids [9,10,11]. Questions, however, remain concerning their sudden proliferation and regionally varying contributions to opioid mortality. Select analyses have characterized the recent, marked proliferation of illicit/synthetic opioids as an independent supply “wave”, whereas others have viewed this as a more dynamic phenomenon in response to reductions in medical opioid dispensing and availability for nonmedical diversion/use, resulting in possible supply shifts or gaps that were increasingly filled by illicit/synthetic opioids [12,13]. Depending on measures used, and while with substantial interprovincial variations, population-level opioid dispensing decreased by up to 50% in Canadian jurisdictions post-2012, similar to overall developments in the US [14,15,16,17,18]. These reductions followed lengthy periods of steep increases in medical opioid availability, and the implementation of multiple system-level interventions (e.g., opioid formulary restrictions, intensified prescription monitoring, revised prescription guidelines, law enforcement) occurring across multiple years post-2010 that aimed to reduce opioid availability and harms (e.g., mortality, morbidity) [19,20,21,22]. These intervention efforts gradually restricted medical opioid flow and supply, and so likely facilitated increasing exposure to alternative illicit/synthetic opioid products by nonmedical opioid users. The proliferating illicit/synthetic opioid products (e.g., fentanyl, fentanyl analogues) have been well-documented to feature high potency/toxicity properties with consequentially elevated overdose and related fatality risks [23,24,25].

Based on possible relationship dynamics between prescription opioid availability and opioid-related mortality, recent exploratory data found the extent of decreases in opioid dispensing were associated with changes in overall levels of opioid-related mortality across the Canadian provinces [26]. Complementing these analyses, based on recent data characterizing opioid mortality profiles, the present study explored potential relationships between changes in opioid dispensing and the specific contributions of illicit/synthetic opioids to opioid-related overdose mortality in Canada.

## 2. Materials and Methods

The specific aim of the study was to assess possible associations between relative changes in medical opioid dispensing and corresponding relative contributions of illicit/synthetic (fentanyl) opioids to total opioid fatalities (2016–2019) across the ten Canadian provinces.

Data used for analyses came from two sources. First, medical opioid dispensing data were derived from previously utilized information on community-based dispensing of prescription opioids from a commercially operated, representative pan-Canadian panel of about 6000 community-based retail pharmacies (compiled by IQVIA, a global health analytics company) capturing the majority of opioid dispensing in Canada [16,27]. With this panel, the dispensing totals for prescription-type medications in Canada are estimated through geospatial projection methodology, as examined by other drug utilization analyses [28,29]. Original dispensing information included summary totals by opioid formulation, strength, and dose, for each of the ten provinces. Based on standard classifications, “strong opioids” (i.e., excluding “weak” opioids, e.g., codeine, as well as methadone due to inconsistent dispensing) were converted into annual defined daily doses/1000 population/day (DDD/1000/day) province-based values for the years 2011–2018 [30,31]. Two a-priori measures were derived for analyses from these opioid dispensing rates. The first was the difference in annual, province-based opioid dispensing (in DDD/1000/day) between 2011 and 2018; the second was the difference between the respective “peak-year” (i.e., highest year) of opioid dispensing (anywhere between 2011 and 2017) and 2018 for each province. The rationale for these two measures was that (a) main opioid control interventions to reduce opioid availability were implemented in 2012 and years following (i.e., with 2011 as the last pre-intervention year), and (b) opioid dispensing changes have varied, e.g., in terms of timing, by province [16,19].

Second, opioid-related fatality (“apparent opioid toxicity deaths”) data for Canada came from recent federal surveillance reports on opioid-related harms [3]. National opioid-related mortality data are based on analyses by provincial coroner services, for which specific methodologies may differ, yet data are federally combined and reported for surveillance purposes [3]. Provincial totals and (crude) population rates of annual (accidental) opioid-related fatalities for the years 2016 to 2019 (the only full years for which such data were available) were extracted. For the mortality indicator of interest, we used the annual proportion (%) of opioid-related fatalities involving “fentanyl” among total opioid-related deaths by province (2016–2019). Given intra-provincial variation in the values yet in the absence of trend-analyses, the respective average percentage values of the provincial fentanyl contribution rates to opioid deaths were calculated and used as a proxy for analyses.

To examine possible associations between the two above-defined measures, we computed the Pearson product moment correlations between the two sets of province-based changes in opioid dispensing ((i) 2011–2018, and (ii) “peak-year” to 2018) and the corresponding provincial average rates of fentanyl-related fatalities (2016–2019). These two approaches for analyses took into account the general timepoints of interventions to reduce medical opioid dispensing (2012), yet furthermore the interprovincial variations in opioid dispensing patterns, including varying timing of “peak-years” and subsequent declines. Statistical significance was set at *p* < 0.05. Correlation statistics were reported, and scatterplots of the correlations generated, with analyses computed using the statistical packages in R [32]. No ethics review was required for the present study due to the anonymous, depersonalized population-level data used for analysis.

## 3. Results

**Figure 1:**
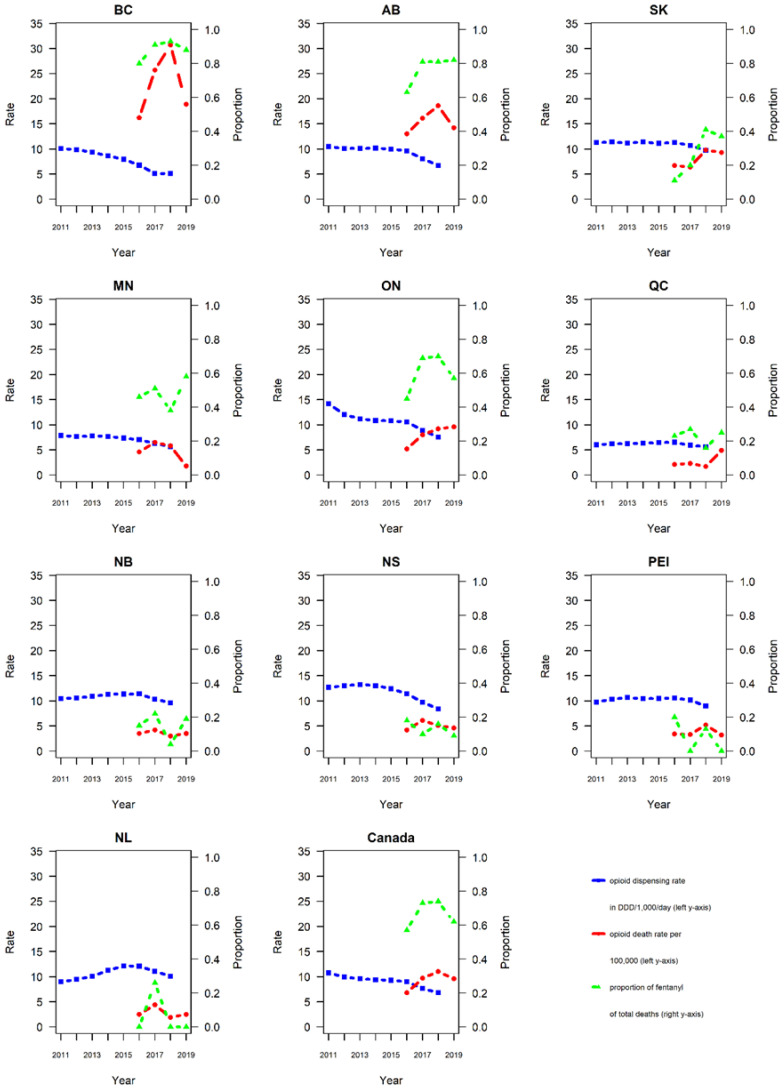
Annual rates of opioid dispensing and opioid-related mortality, and proportion of fentanyl among opioid-related deaths, for provinces and Canada total (for abbreviations of provinces see Table 1).

Data on annual opioid dispensing rates, total opioid-related mortality, and relative contributions of fentanyl to opioid-related deaths, by province and Canada total, for the respective data periods are presented in Table 1 and visualized in Figure 1. Table 1 also includes information for the provincial acronyms.

In 2011, ON had the highest rate in strong opioid dispensing (14.2 DDD/1000/day), while QC had the lowest rate (6.0 DDD/1000/day). Regarding changes in opioid dispensing, ON had the largest reduction (−6.7 DDD/1000/day), whereas NL had the smallest reduction (+1.1 DDD/1000/day increase) between 2011 and 2018; for the “peak year” to 2018 measure, ON had the largest reduction (−6.7 DDD,1000/day), whereas QC had the smallest reduction (−0.9 DDD/1000/day).

Provincial opioid-related fatality rates ranged from 2.1/100,000 in QC to 16.2/100,000 in BC in 2016; corresponding rates ranged from 1.8/100,000 in MB to 18.8/100,000 in BC in 2019. All but one province (MN) reported either stable or increasing overall opioid-related mortality levels between 2016 and 2019.

Province-based percentages of fentanyl-related fatalities among total opioid-related mortality varied. PE had the smallest average percentage (8%; annual range 0–20%); BC had the highest percentage (88%; 80–93%) of fentanyl-related fatalities among total opioid-related fatalities between 2016 and 2019. Three provinces (BC, AB, ON) had an average rate of fentanyl-related mortality >50%, five provinces (QC, NB, NS, PEI, NL) had an average rate <25%.

**Figure 2:**
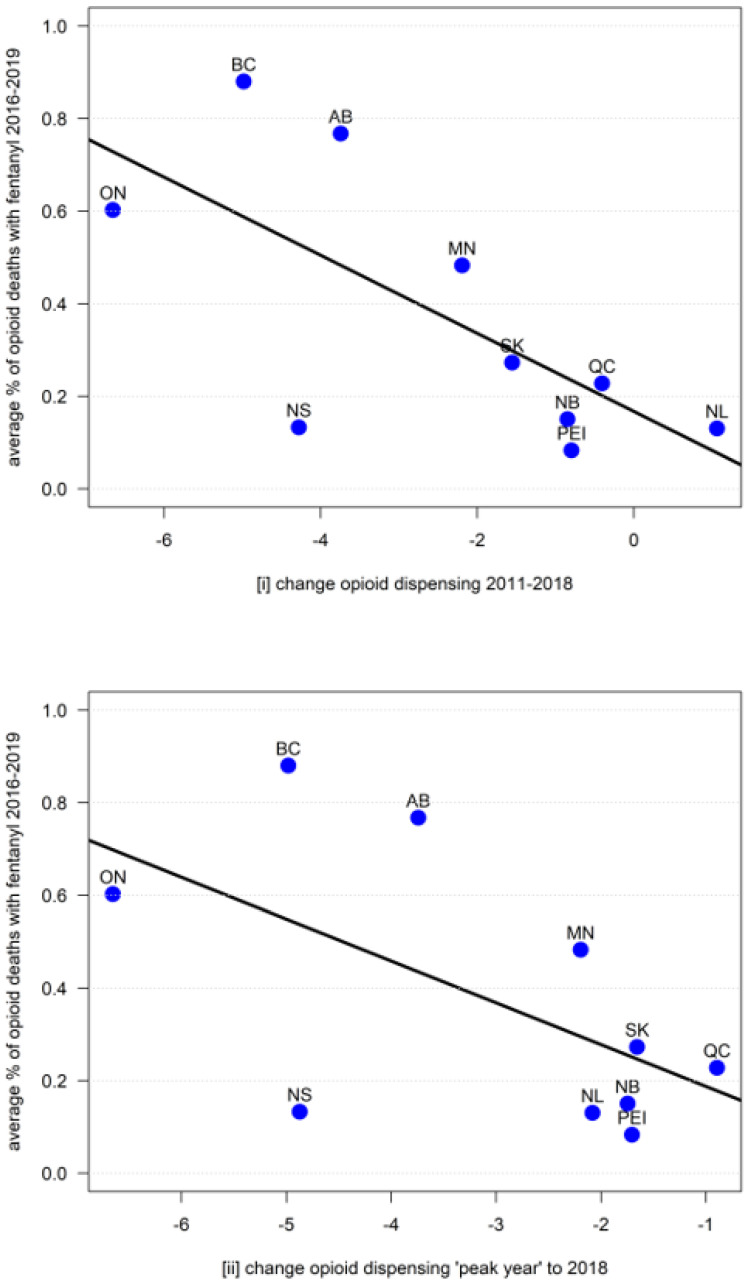
Correlations between opioid dispensing change ((**i**) 2011 to 2018 and (**ii**) “peak-year” to 2018) in DDD/1000 population/day and average percent of opioid deaths involving fentanyl (2016 to 2019), by province in Canada (for abbreviations of provinces see Table 1).

The Pearson’s product moment correlation between (i) the provincial changes in opioid dispensing (2011–2018) and the average percentage of fentanyl of the opioid-related fatality total (2016–2019) was −0.70 (*t* = 2.75, df = 8, *p* = 0.03); the corresponding correlation between (ii) the province-based changes in opioid dispensing (peak year to 2018) and the average percentage of fentanyl of the opioid-related fatality total (2016–2019) was −0.59 (*t* = −2.06, df = 8, *p* = 0.07), indicating significant and near-significant results, respectively (see Figure 2 for corresponding scatterplots).

## 4. Discussion

We presented evidence of (near-)significant correlations between the rates of decreases of medical opioid dispensing (in DDD/1000/day) post-2010 and the relative contributions of fentanyl to total opioid-related fatalities (2016–2019) in the Canadian provinces.

Opioid dispensing reductions considerably varied across provinces, from no/minimal decreases to reductions up to 50% [15,17]. Similarly, contribution rates of fentanyl to total opioid-related mortality varied: while half of the provinces indicated consistently minor (<25%) average contribution rates, three reported substantive (>50%) majority rates. The provinces with the highest relative fentanyl contributions to mortality showed the largest reductions in opioid dispensing. Hence, our results suggest that the larger the reductions in medical opioid dispensing, the higher the contribution of fentanyl to the opioid-related fatality total in a province. The strength and consistency of the correlations are notable, given the small number of datapoints (10 data pairs/provinces) available for analyses. These results complement findings of previous examinations that found provincial levels of reductions in medical opioid dispensing to be associated with changes in overall opioid mortality levels [19,26]. While exploratory in nature, the present findings may support the possibility that recent reductions in medical opioid dispensing, following various system-level interventions to reduce opioid-related harms, may have led to supply shifts or gaps in opioid availability, especially for nonmedical use, resulting in increased illicit/synthetic opioid exposure and related overdose fatalities across Canada.

Illicit/synthetic opioid products (i.e., fentanyl and fentanyl analogues) increasingly proliferated in the nonmedical opioid supply, and came to make growing contributions to opioid-related mortality in Canada over the past decade, similar to US experiences [6,8,12,25,33]. Decreasing supply of medical opioids—and correspondingly reduced availability for nonmedical opioid use—may have led (existing or new) nonmedical opioid users to increasingly resort to and rely on illicit/synthetic products (fentanyl or others) as are documented to be highly potent, and toxic towards elevated risk for overdose (death) [10,11,34]. This aligns with individual-level observations of transitions from prescription to illicit/synthetic opioid use, and increased overdose risk, for different user populations [35,36,37]. Other data have documented increasingly restrictive opioid control environments to facilitate a “drying up” of medical opioid supply, pushing nonmedical opioid users into growing use of, and exposure to, risky, illicit/synthetic (e.g., fentanyl) opioids [38,39].

On this basis, policy efforts to restrict high levels of medical opioid availability may have exceeded efforts to reduce population-level demand for nonmedical use, increasing discrepancies between opioid-related supply and demand [40]. Increases in illicit/synthetic opioid use and related fatalities may have occurred as an unintended adverse consequence of these dynamics [6,26,41]. Similar adverse “substitution effects” from supply control efforts towards more hazardous substance types have been documented elsewhere [42].

For methodological limitations, while DDD are a generally good measure for comparative opioid consumption estimates, their accuracy is limited [17,43]. Community-based opioid dispensing data do not include dispensing from other sources (e.g., hospitals, Internet) which, however, involve relatively minor amounts. The federal opioid mortality data examined is based on provincial coroners’ toxicological analyses, involving possibly different analytical standards and practices. Not all fentanyl identified in toxicological analyses is necessarily illicit/synthetic; illicit/synthetic opioids other than fentanyl or fentanyl analogues secondarily contribute to opioid fatalities [3,44]. Differential patterns of fentanyl contributions to opioid mortality could be influenced by different factors, for example, regional/geographic, economic effects, or the direct or indirect impact of interventions targeting opioid use and related risk (e.g., prevention or treatment measures) [45].

## 5. Conclusions

In sum, this study presented additional exploratory evidence that the extent of medical opioid dispensing reductions appears to (negatively) correlate with relative contributions of fentanyl to total opioid fatalities across Canada. These dynamics have translated into overall adverse effects for public health, involving substantial increases in the total of opioid-related deaths in Canada, especially including the larger provinces comprising >80% of the population. Emerging opioid supply shifts from pharmaceutical-grade opioids towards illicit/toxic opioids may have reduced select harm outcomes related to medical opioids (e.g., iatrogenic addiction), yet may have facilitated increasing fentanyl-related mortality for distinct policy trade-offs. Given the present study’s exploratory nature, these overall dynamics should be further examined through other or expanded data (e.g., considering possible covariates) and/or methodological approaches. Given the limited understanding of the opioid crisis’ supply dynamics, especially on health outcomes [12], our findings warrant consideration for ongoing public health-oriented interventions and policy development. As long as substantive demand for nonmedical opioid use exists amidst an extensive supply of illicit/synthetic opioids, expanding needs-based opioid pharmacotherapy options and “safer supply” interventions for at-risk users form crucial measures towards reducing excessive opioid mortality alongside other interventions [46,47,48].

## Figures and Tables

**Table 1:** Changes in annual opioid dispensing rates (2011–2018 and “peak year” to 2018), annual opioid-related mortality rates, and proportion of fentanyl-involved mortality among total opioid-related deaths (2016–2019), by province and Canada total.

	Annual Values and Change in Opioid Dispensing (DDD/1000/Day)		Annual Number (Crude Rate Per 100,000) of Opioid-Related Mortality, 2016 and 2019		Range and Average of Annual Percentage of Opioid-Related Mortality Involving Fentanyl, 2016–2019	
Province	2011–2018	“Peak Year” to 2018	2016	2019	Range	Average
**BC**	−5.0 (10.1–5.1)	−5.0 (10.1–5.1)	789 (16.2)	959 (18.9) ^±^	80–93%	88%
**AB**	−3.7 (10.5–6.7)	−3.7 (10.5–6.7)	547 (13.0)	620 (14.2)	63–82%	77%
**SK**	−1.6 (11.3–9.7)	−1.7 (11.4–9.7)	76 (6.7)	109 (9.3)	11–41%	27%
**MN**	−2.2 (7.9–5.7)	−2.2 (7.9–5.7)	61 (4.6)	24 (1.8)	38–58%	48%
**ON**	−6.7 (14.2–7.6)	−6.7 (14.2–7.6)	726 (5.2)	1397 (9.6)	45–70%	60%
**QC**	−0.4 (6.0–5.6)	−0.9 (6.5–5.6)	173 (2.1)	414 (4.9) ^±^	16–27%	23%
**NB**	−0.8 (10.5–9.6)	−1.7 (11.4–9.6)	27 (3.5)	27 (3.5)	4–22%	15%
**NS**	−4.3 (12.7–8.4)	−4.9 (13.3–8.4)	40 (4.2)	45 (4.6)	9–18%	13%
**PEI**	−0.8 (9.7–8.9)	−1.7 (10.6–8.9)	5 (3.4)	5 (3.2)	0–20%	8%
**NL**	1.1 (9.0–10.1)	−2.1 (12.1–10.1)	13 (2.5)	13 (2.5)	0–26% *	13%
**Canada**	−4.0 (10.8–6.8)	−4.0 (10.8–6.0)	2466 (6.8)	3617 (9.6)	57–74%	67%

BC = British Columbia, AB = Alberta, SK = Saskatchewan, MN = Manitoba, ON = Ontario, QC = Quebec, NB = New Brunswick, NS = Nova Scotia, PEI = Prince Edward Island, NL = Newfoundland and Labrador. * data suppressed for 2016 and 2019. ^±^ Includes deaths related to all illicit drugs including, but not limited to, opioids and stimulants.

## Data Availability

The datasets analysed for the present study was based on information from a commercial database (IQVIA Canada’s Compuscript) on medical pharmaceutical (including opioid) prescriptions in Canada, available on request and under license from the source. National opioid-related mortality data used is publicly available from the Public Health Agency of Canada through its public surveillance and information database (Available online: https://health-infobase.canada.ca/substance-related-harms/opioids-stimulants/, accessed on 24 June 2021).
